# Psychologists experience of cognitive behaviour therapy in a developing country: a qualitative study from Pakistan

**DOI:** 10.1186/1752-4458-4-2

**Published:** 2010-01-28

**Authors:** Farooq Naeem, Mary Gobbi, Muhammad Ayub, David Kingdon

**Affiliations:** 1 Lahore Institute of Research and Development, Lahore, Pakistan; 2 Medicine, Health and Life Sciences, University of Southampton, Southampton, UK; 3 Psychiatry of Learning Disability, Tees Esk & Wear Valley NHS Foundation Trust, Darlington, Durham, UK; 4 University Department of Psychiatry, Royal South Hants Hospital, Southampton, UK

## Abstract

**Background:**

Psychological therapies especially Cognitive Behaviour Therapy (CBT) are used widely in the West to help patients with psychiatric problems. Cognitive Behaviour Therapy has an established evidence base for the treatment of different emotional disorders. In spite of these developments in the developed world, patients in most developing countries hardly benefit from non pharmacological interventions. Although a significant number of psychologists are trained in Pakistan each year, psychological interventions play only a minor role in treatment plans in Pakistan. We conducted interviews with psychologists in Pakistan, to explore their experiences and their views on "providing CBT in Pakistan". These interviews were conducted as part of a project whose focus was to try to develop culturally-sensitive CBT in Pakistan.

**Methods:**

In depth semi structured interviews were conducted with 5 psychologists working in psychiatry departments in Lahore, Pakistan.

**Results:**

All the psychologists reported that psychotherapies, including CBT, need adjustments for use in Pakistan, although they were not able to elicit on these in details. Four major themes were discovered, hurdles in therapy, therapy related issues, involvement of the family and modification in therapy. The biggest hurdles in therapy were described to be service and resource issues.

**Conclusions:**

For CBT to be acceptable, accessible and effective in Non Western cultures numerous adjustments need to be made, taking into consideration; factors related to service structure and delivery, patient's knowledge and beliefs about health and the therapy itself. Interviews with the psychologists in these countries can give us insights which can guide development of therapy and manuals to support its delivery.

## Background

Cognitive behaviour therapy has become established as the most researched and effective psychological intervention for a wide range of mental disorders including depression, anxiety, Obsessive Compulsive Disorder, Bulimia, Personality Disorders and Schizophrenia within the developed countries [1-5]. Although, there have been limitations on availability, National Clinical Guidelines are leading to therapy being made available across the developed world (for example clinical guidelines for the treatment of depression, by the National Institute of Clinical Excellence (NICE), UK http://www.nice.org.uk.

Currently, CBT is only rarely available in the Non Western, developing countries. There is some evidence to suggest that CBT might be effective in treating mental health problems in developing countries [6-8]. The cost of medicines in the developing world might inhibit effective treatment availability [9]. Due to the low cost of available human resources, psychological help may be less costly to provide with additional therapeutic benefits and as an alternative to medication. Most patients have to buy medicines privately in Pakistan, where price of the cheapest SSRI is Rs. 20 (0.25 USD). The cost of a session of therapy by a psychology graduate is approximately Rs. 100 (1.28 USD) (based on a salary of 10,000 Pakistani Rupees per month for a psychology graduate). It is therefore important to study factors which have prevented CBT from gaining popularity in Pakistan.

It has been suggested that CBT might need cultural adaptation before its application in non western cultures. This is due to the multiple issues which can have an effect on application of CBT in non western cultures [10-13]. Experienced psychologists modify therapy for the individual needs of patients in Pakistan taking into considerations cultural and religious factors, like elsewhere. For example, Murray [14] has pointed out that the psychologists working in Pakistan use religious practices as part of the therapy.

Most universities in Pakistan, whether private or state run, have a psychology department (Twelve such courses are being run in only Lahore, capital of the province of Punjab). There are approximately 60 state run universities in Pakistan http://www.pakrang.com/education/universities-list.htm and a higher number of universities within the private sector. These departments offer both bachelor and masters programmes. However, to practise as a psychologist, it is necessary to do a university based post graduate diploma. There are currently five institutes that provide postgraduate diplomas in Pakistan. The training is provided using an eclectic approach. While students have access to local hospital patients, some institutes also have their own counselling centres, which are mainly attended by the students. There is no national institute for accreditation or registration. Psychologists gather under two professional bodies in Pakistan. The Pakistan Psychological Society has a membership of nearly 500, while the Pakistan Association Of Clinical Psychologists has a membership of nearly 75. Most psychologists join Non Government Organisations after postgraduate qualifications; however, a small number also joins the psychiatry departments of state run hospitals. These departments have very limited number of posts for psychologists. None of the hospitals in Pakistan has an established psychology department. Psychologists work as part of the medical team. Not all of these psychologists have post graduate qualifications. Psychologists also work as generic mental health workers in psychiatry departments where they are involved with history taking and assessments in the outpatient clinics. Outpatient clinics in Pakistan are like drop in centres in the UK and any patient from any part of the country can present himself to the outpatient department for assessment.

The current study was performed as part of a "Developing Culturally-Sensitive CBT Project" in which we are developing methods to modify CBT for psychosis in the UK [15] and CBT for depression in Pakistan. This latter project involved a series of studies whose aim was to determine what factors should be taken into account so as to develop and refine a CBT manual to treat depression. The study received ethical approval from local ethics committee. The study comprised interviews with patients who were currently receiving treatment for depression; psychologists who regularly managed patients with depression; and group discussions with University students. Interviews with patients were conducted to elicit their beliefs about depression and mental illness, their knowledge of depression and its treatment especially non-pharmacological intervention. Group discussions with students attending Masters Courses in a University were conducted to discover whether the students' concepts relating to CBT and western psychotherapy were consistent with their, personal, family, social and cultural and religious values [16].

This paper reports the qualitative findings and implications of the information received through the psychologists' interviews. The study sought to identify the factors which might need modification or consideration in the use of CBT in Pakistan through eliciting the experiences, perceptions and expertise of the local psychologists.

### Aims and purpose

The aims of this study were to;

(1) Elicit psychologists' experience of CBT and/or their experiences of clients with depression for whom CBT might be an appropriate therapy; and

(2) Identify factors that should be taken into account when developing CBT and the accompanying training manual for use in Pakistan.

The purpose of the interviews was to;

(1) Ascertain how the psychologists help their patients with depression

(2) Gain a comprehensive understanding as to the kinds of CBT techniques therapists use that are acceptable to their patients

(3) Discover whether the therapists employed any techniques that had been developed from local traditions.

## Methods

One-to-one semi structured interviews were considered to be more effective in gaining information from psychologists rather than a survey, because this topic was an unexplored area in the context of Pakistan. We wanted to engage in a meaningful dialogue with the psychologists which would address some pre-determined themes, but would also be responsive to issues that were raised in discussion. This would also enable the exploration of difficult or contentious issues (e.g. do patients in Pakistan present differently to those from other countries, and how this is taken into consideration when applying techniques from the western psychotherapy?). Individual interviews would also enable clarification of any ambiguities or language nuances.

### Areas of interest

Drawing on the literature review, the lead author's (FN) experiences as a practitioner in Pakistan and data from other countries, a list of important issues to guide the interview was prepared, this consisted of the following;

1. Background of the psychologist (name, age, training, years of experience, area of training)

2. Their area of work (adult/child, individual/group etc.)

3. Their typical patient load and the proportion of patients who suffer from depression

4. What are the other problems with which patients present

5. How many sessions are usually provided to patients in routine therapy?

6. The attendance and attrition rate from therapy sessions

7. Whether they find it easy to understand western psychotherapy techniques specially those from cognitive therapy

8. Which techniques are used most frequently?

9. Whether patients find some techniques unhelpful

10. The techniques patients prefer

11. Patients' expectations of therapists

12. Elements of therapy, e.g. the role of the family and community

13. Distinctions in presentation of depression and anxiety

14. Whether there is a need to modify CBT techniques for use in Pakistan and if so, how?

### Sample

The intended population of psychologists was those working in the psychiatry departments of the teaching hospitals in Lahore. We sought psychologists who preferably practised CBT. However, it was discovered that they were only trained in Rational Emotive Behaviour Therapy. Since there are many similarities between CBT and REBT we decided to proceed with the interviews. Psychologists reported that they were aware of the basic concepts of the CBT. An intermediary provided a list of all the psychologists from Lahore, they were approached by telephone. All those who were contacted agreed to participate. The final interview sample was drawn from those who provided consent. It was estimated that the sample size was likely to be in the region of 5-10 participants or until data saturation was reached and no new themes emerged. In effect, after 5 interviews and through discussion with the qualitative research supervisor (MG) we found that no new themes were being generated and the data was saturated. The reason for this could be that all the psychologists received their postgraduate qualifications from the same institute and had similar clinical experiences and were working in large city teaching hospitals in the state run health service.

### Interview process

An interview guide that incorporated the areas of our interest was developed to ensure consistency of approach. Psychologists were asked about their experience of therapy with patients focusing upon those with depression attending the state run health service. However, it became apparent that therapists also referred to patients in the private sector and data concerning this was also gathered when appropriate. The study was conducted in two parts. In the first part we conducted in depth interviews of a sample of the psychologists (N = 5). All the psychologists were interviewed (by FN) in their hospitals. The audio-recorded interviews lasted between 30 to 60 minutes using the interview guide, commencing with details about the psychologist and their practice. The interviews were conducted in English since English is widely spoken by the health professionals in Pakistan. Anonymity and confidentiality were assured. The interviews were conducted between February and April 2007. During the second part of the study, following transcription, the interview scripts were returned to the psychologists for comment, verification and for clarity with respect to queries that arose from the analysis stage. In addition, some individuals were contacted by phone to clarify points and emergent themes that arose from the initial transcriptions or analysis. The verbatim interviews were analysed by MG, MA and FN for emerging themes. The themes were then converted into codes. In the final analysis these were built into categories. Transcripts were sent again to the psychologists and they were asked to identify any point they did not agree or wanted to comment upon. In an attempt to triangulate and judge the generalisability of the data, the interview transcripts were reviewed by seven [7] psychologists from the original list working in Lahore, Pakistan (different from those who were initially interviewed). Their comments and observations were noted if different from the previous ones. However, no major differences emerged and this confirmed that the data were saturated with respect to the research aims.

### Analyses

Interviews were analyzed using thematic content analysis. We started transcribing interviews as soon as the process of the interviews started. The interviews were transcribed by the first author (FN). The interviewees were ascribed numbers which were used in the transcription and report. Three of the authors FN, MG and MA closely read transcriptions as and when they became available, identifying topics of interest (open codes) either because they already existed in literature (for example, somatic presentation of illness) or they were important because of the areas we wanted to explore (for example, effect of services structures on therapy or literal translation of the concepts of therapy). MG provided supervision and support through telephone throughout the study. We also held regular meetings in which emerging themes, concepts and conflicts were discussed. These separate readings were compared and discussed in details throughout the period of study. This helped us to construct a synthetic set of codes to guide not only the next interview but also the analysis of the transcripts. This process was repeated, thus modifying the working codebook till a final set of codes was obtained. The process stopped when saturation point was reached and we realized that no new themes were emerging. Finally the data was reorganized into wider themes (for example hurdles in therapy) and categories (for example homework) and written for this paper.

## Results

The five psychologists who were interviewed were all females. This is due to the fact that psychologists are mostly females in Pakistan. All of them were trained in Rational Emotive Behaviour Therapy (REBT), although they reported being aware of other CBT techniques. While they all worked mainly in the state run health service, three also worked in the private sector in the evenings. They all worked with male and female adult patients. Their experience ranged between 3 to 15 years. Each one had a postgraduate diploma.

Psychologists reported that patients come with psychiatric problems as well as emotional and social problems. The common diagnoses with which patients present include anxiety and depression (nearly 60-70% of the patients have a mixture of anxiety and depression). Patients also present with conversion disorder, family conflicts, broken love affairs, bereavement, and OCD.

### Hurdles in therapy

#### Service issues

The health service is poorly structured and specialist services are only limited to the big cities. In addition to seeing patients in the outpatient services, psychologists also receive referrals from psychiatrists, through Accident &Emergency and other departments in the hospital and 'self referrals' from patients. Patients attending the state run health service are usually poor, not well educated and come from remote places (e.g. one patient who travelled from Kashmir to Lahore for treatment took two days to arrive). According to one psychologist between 70-80% of the patients come from outside Lahore city. Some of the patients who see psychiatrists or psychologists in private practices might be well educated and not very poor. However it appeared in later discussions that the outcome of the therapy for those, who live locally, are educated and come from effluent background is not entirely different from the rest. One psychologist thus described the service structure,

*(We) cater our services to poor people who come from different areas of (the province of) Punjab. They are usually illiterate and belong to the (lower) or lower middle (social) class. So, how can you give them therapy? In my private practice I (sometimes) deal with educated people (I_1). They are also not keen on therapy(I_3). And there is no referral system (I_2). We have to see patients in outpatient department for screening (I_5)*.

Due to the small number of psychiatry departments, patient load is very high. The number of psychologists working in the health system is even smaller than the psychiatrists. This obviously means that the psychologists have to see a higher than usual number of patients for therapy. One psychologist reported that,

*" in the Out Patient Department we usually see around 20 to 30 patients (I_1) *whereas another had to deal with *" 06 or 07 patients per day (I_2)" *for psychological therapies.

We should keep in mind that an average working day consists of 5 hours. So it is hard to imagine how much time each patient gets and how so many patients are managed. It is not surprising that a session can be as short as 15 minutes. Only a small proportion of patients (less than a third according to most psychologists) returned for follow up appointments, with most patients attending for only 2 or 3 sessions. The drop outs are understandably higher for those coming from outside Lahore; however, they also described other factors responsible for high attrition,

*For illiterate patient coming from outside Lahore therapy lasts over hardly one or two sessions (I_2).Apart from distance, socioeconomic status, female gender and patient not being psychological minded are other reasons for drop outs (I_3)*. *Patients stop coming as soon as they are symptom free (I_4)*. *Doctors also don't refer them properly, Psychiatrists usually say to patients "go to the Psychologist and they will give you relaxation therapy", they [the doctors] do not even know anything about cognitive therapy (I_2)*.

#### Dealing with somatic complaints

Patients present with different diagnoses, but mostly present with physical symptoms. We wanted to know how therapists deal with these somatic complaints, however, none of the psychologists were able to elicit this further.

Sometimes it's depression due to for example, some type of family problem, marital conflict, broken love affair or the death of a loved one (I_3). But almost all our patients present with somatic complaints. It is difficult to tell them that they have no physical problem but only psychological problem, which can be helped by psychotherapy (I_1).

#### Pills and psychotherapy

It was typically mentioned that patients always receive psychological help in combination with medical treatment, for example *"as far as the psychological sessions are concerned, they are always accompanied by the medical treatment" (I_3)*. As one psychologist noted *"we are a pill oriented society" (I_1)*. This was explained by a colleague, *" I think that the patients are more interested in medicines because "they feel good with medicines"(I_4)*.

#### Homework

One important aspect of CBT is its emphasis on homework. Therapists working in the west find adherence can be a problem. However, this is particularly a difficult area in Pakistan. Some psychologists suggested that it might be due to illiteracy. However, psychologists working in the private sector with educated patients also reported that patients were not keen on doing the home work, as these psychologists explain,

*Not all the patients follow home work assignments, may be only up to 40% do (I_3)*. *Only one third patients do the homework, and they also don't do it for all the sessions (I_2)*.

#### Patient's expectations from mental health system

Patients knowledge of and their expectations from the mental health system are pivotal in their help seeking behaviours, engagement, compliance and follow up with the therapy. As some of the psychologists speculated, it is possible that people probably do not expect psychological therapies in a medical system,

May be they are not expecting this kind of therapy. They are expecting only medicines from here.... And when we talk to them, they feel it is only chatting although we are very purposeful. They feel that this is like a Gup Shup (chit chat). And they are not able to perceive the underline meanings, although we are very meaningful (I_5).

#### Literal translation does not work

Psychologists discussed how they find it difficult to explain the cognitive errors. They reported giving long descriptions to the patients in order to explain cognitive errors and they used literal translations of the cognitive errors. For example most psychologists translated black and white errors into its literal Urdu or Punjabi translation, which does not translate into a phrase which is readily understandable. Although when you say black and white thinking to an English speaking person he will have a good idea of what it means. Literal translations of terminology can pose additional problems, such as,

*Sometime people do not understand the cognitive errors. When I use the term negative thinking they will say, "No, No, we have no negative thoughts". I think this is because they don't know what the negative thoughts are. They think negative thoughts are very bad. It is something evil (I_2)*.

#### Beliefs about illness

Involvement of non-medical healers was described to be a hurdle in therapy. Being aware of patients' beliefs about the cause of the problem can be helpful. It appears that some patients believe in non-medical causes like magic and they seek help from faith healers or religious healers. One psychologist tried to clarify the difference between faith healers and religious healers,

*Patients usually go to see the religious healers. They also go to see the spiritual healers, but mostly they see religious healers. The reason they see the religious healers is because they believe their illness is due to Jadu (Magic) (I_1)*.

### Issues related to therapy

#### Assessment

The process of assessment does not seem to be very different from that used in the west. Most psychologists said that they include the family in assessment. Assessment consists of history taking and formulating a management plan. Sometimes assessment includes Psychometric testing. They focus on the patient's problems and later on problems are addressed on a cognitive level and emotional level. However, the first step in therapy remains a careful assessment, as these psychologists describe;

*Patients tell us about their problems and they usually have a diagnosis of depression or anxiety and they mostly present with physical complaints or with conversion, so the first step is to assess them(I_1)*. *We look into the problems and sometimes make a list of patient's problems or complaints (I_5).*

#### Commonly used techniques

Psychologists described frequent use of common Cognitive Therapy and Problem Solving Therapy techniques, for example,

*We commonly use CBT techniques such as, identifying and teaching on cognitive errors, monitoring mood, cognitive restructuring, use of diaries to identify and change thoughts and working on irrational beliefs (I_3)*. *Monitoring mood is a very good technique (I_4)*. *We also provide coping statements and for certain problems build a list of problems. We also use problem solving, social skills training, building coping strategies, activity plans and assertiveness training (I_5)*.

#### Structure and content of sessions

The number and length of sessions varies from patient to patient. We have already mentioned low rates of engagement and high rates of drop out. Most psychologists said they usually plan 12 sessions. However they described that it is not always possible to follow a structured session as these remarks indicate,

*A therapy session can last between 15 minutes to an hour or even more (I_5)*. *As you can imagine it is not always possible to discuss the formulation with the patients (I_1)*.

#### Normalizing techniques

Judicious use of humour can facilitate therapeutic process and improve rapport. This can be quite useful when sharing a personal experience or to normalize an experience.

*Sometimes we use humour with them. Let me give one example, one of my patient said that she had been scolded by mother in law and I said every mother in law is like that, you are not on your own (I_1)*.

### Techniques which patients find helpful

A typical statement was that at the start patients find behavioural techniques easier to follow. Similarly building strength and difficulties and using prompt cards are easy to use by the patient.

*On a behavioural level_ if a person is depressed I will help him to improve his activities by using behavioural techniques (I_1)*. *"for a patient who comes with anxiety I use relaxation exercise to help him or her. Most patients only need relaxation techniques (I_3)*. *when they come with problems, I always use problem solving and they find it very helpful (I_4).*

#### Style of therapy

Psychologists explained that the style of therapy in Pakistan is more instructional than collaborative. This could be due to the fact that the patients ask for direct advice and they like suggestions. Our first interviewee said,

*Our patients like us to advise them on different issues. They typically come and say "Doctor please tell me what should I do now. This is what they expect from us (I_1)*.

#### Involvement of the family

In the absence of a health system family and friends is the only network of support patients can access in Pakistan. However there are problems involved with family as well, as most psychologist described, exemplified by this, *In Pakistan the family is too much involved with the patients, and sometimes it can be a big problem. However, family can also be very helpful in different ways (I_2)*. *I usually go for independent session with the family as well, and sometime family does not want to disclose anything about the patient (I_3)*.

### Modifications in therapy

Psychologists try to tackle the hurdles by doing whatever they feel is suitable. However, it was difficult to get any concrete information regarding the changes that have been made and are widely used or are acceptable in the use of therapy. These examples demonstrate some of the adaptations that the psychologists have developed,

*We focus on home work and discuss the importance of home work with our patients. But they do not do it. So I give them homework during the therapy session and this will be a kind of punishment (I_2)*. *I usually take the patient's educational and the personal background in mind when giving therapy (I_4)*. *We arrange sessions variably. The gap between two sessions can vary between 2 to 4 weeks (I-1)*.

We tried to explore this part of the interviews in detail. We were interested in exploring modifications in techniques. Although, we were unable to identify common trends or obvious changes in techniques, all the psychologists agreed that the therapy in its current form is not applicable. They stated that changes need to be made in therapy to suit the needs of the patients in Pakistan. They all reported that they have to change therapeutic techniques according to the personal requirement of the individual. However, we were unable to obtain further information on how this was achieved.

## Discussion

This is the first study to explore psychologists' experiences with the use of psychotherapy and in particular CBT, from a developing country. Since psychologists in Pakistan are not trained in CBT in Pakistan we had to interview psychologists who were trained in REBT. Earlier in our work we realised that it was a difficult task to carry out. Our impression was that participants were not very comfortable when admitting to a lack of knowledge on some subject. This has obvious implications for interview studies in Pakistan. However, we gleaned some information regarding their experiences of psychotherapy. We also gained some understanding of how the psychological services work.

We started an investigation into the content and style of therapy and quickly discovered that the broader service and resource issues heavily impinge on the style and content of the therapy. The issues are service structure, delivery and organization (distance, finance, knowledge, and referral path), biological orientation of mental health services and patients beliefs about mental health and health system. As far as we are aware no serious attempts have been made at institutional level in Pakistan to adapt therapy or to improve access to psychological interventions. It seems that therapy is more of a ritual rather than an attempt to change the situation.

Mental health facilities are limited to only a few big cities in Pakistan. Patients come from far off places to see the mental health professionals in these centres. Most of the patients refer themselves to the specialist services. Patients have to travel for long distances and often they come as a family and might even bring friends with them. The Mental health system is very biological in its treatment approach. There are no written protocols about the referral process. Psychologists also help the psychiatrists in their clinics as generic mental health workers. The number of patients seen varies from day to day and across the country. Sometimes the number reaches more than 100 per day.

While everyone acknowledges that most patients come from a significant distance away, there is no mechanism in place and no strategies in practice to make a 'triage' decision. For example, which patients should receive psychotherapy, which ones should be rejected, and how patients coming from a distance could be helped psychologically. It is understandable that most people coming from distant places might not be able to attend regular sessions of therapy. It is therefore not surprising that they don't turn up for follow up. However, psychologists also admitted that even those who come from the same city drop out after a few sessions.

Women are more likely to drop out than men. Yasmin Farooqi [17] has reported that more men than women patients attend traditional healers in Pakistan. Does this apply to psychotherapy as well? Women are usually dependent on men to be brought to the hospitals. They at least have to seek permission from men. Men on the other hand can travel more, are in control of finances and are more educated than women. In the end it could be possible that men are more prone to therapy because psychologists are usually female. Poverty, illiteracy and poor referrals were described as other possible reasons for poor follow up. The observation that patients stop attending therapy as soon as they are symptom free has a logical appeal. The state does not help the diseased or the disabled and people have to return to their work as soon as they can to earn their livings. Some psychologists arrange follow up at longer gaps to allow patients coming from distant places to be able to return. However, lack of a mechanism to support them between the two appointments and no written, audio or video material and lack of interest in home work all possibly minimize the impact of this useful strategy.

Psychologists see high number of patients for therapy. It is hard to imagine how they manage this number along with other duties. This certainly can have an effect on the quality of the therapy patients receive as well as cause stress for the therapists. The typical session starts with assessment and if we take into consideration the high rates of dropout, it possibly ends with assessment! A structured format is not followed and formulation is possibly not always drawn.

Psychiatric patients in Pakistan present with somatic complaints. High prevalence of somatic symptoms in Asian cultures is widely known. Somatic presentation possibly explains why pills are more acceptable and the 'pill prescriber' is held in high esteem. But it is not a straightforward issue. One study from Karachi, Pakistan has reported that patients with physical complaints (musculo-skeletal problems, headache, high blood pressure & angina, headaches, fever, jaundice, Diabetes Mellitus, Epilepsy, Gastro-intestinal problems, Eye diseases, Asthma and Pneumonia, Sexual problem) go to spiritual healers who do not prescribe them medicines [18]. A psychiatric patient presenting with physical symptoms and being treated with medicines might also mean less stigma attached. However, all it means in terms of a trial of cognitive therapy is that we should keep in mind that taking medicines has certain implications which are beyond the boundaries of psychopharmacology. These are traditional, cultural, economic, organisational, political and even financial possibly for the patient but definitely for the prescriber.

Psychologists described patient’s consultations with spiritual and faith healers to be a problem. It was reported that patients see them because of beliefs about magic etc. Some of these beliefs, for example; belief in jadu (magic), saya (possession) and religious or spiritual causes of illness are common. The fact remains that most patients attend faith healers, spiritual healers and traditional healers [19,20]. Knowledge of the patient's knowledge about and expectations from mental health system are important considerations. Interestingly people follow faith healers for life. They ask them to make major changes in life style, read verses or lot of other things in between the meetings. This is similar to homework. The question remains why people don't go back to mental health practitioners?

Although problems with "home work" are frequently reported by therapists in the west, it seems to be one of the biggest problems in Pakistan. Some therapists reported that illiteracy is a possible reason, while others noted that even educated patients don't like to do written home work. It is difficult to know what they meant by literacy? Home work involves writing. However, we have observed that people in general do not like to read and write in Pakistan. Verbal commitments are still respected. This is seen commonly when people are getting into a contract, for example, dealing with a shop keeper, a mechanic or a tailor.

Language is the main tool which is used to deliver psychotherapy. Bibliographical material in English language is provided to patients during therapy. English is the official language of Pakistan. Urdu is the national language of Pakistan. Although, nearly 80% people can speak Urdu in Pakistan majority of people don't speak this language as their mother tongue. The languages distinctively spoken in Pakistan include Punjabi (45%) (with 8 distinct dialects), Sindhi (15%), Pashto (15%) Siraiki (10%) Balochi (4%) and Other languages (11%), which include Burushaski, Shina, Khowar, Kalash and Wakhi http://www.wikipedia.com. Even the very educated may not be able to understand psychological terminology in English or even Urdu. This leads to yet another important issue. While translation of medical or psychiatric information for patients might not pose many difficulties, literal translation of psychotherapeutic concepts for use in a non western culture might cause certain problems. One such example is the translation of the term "negative thoughts", which psychologists reported people don't like. Similarly, literal translations of cognitive errors were also described to be important hurdles in therapy.

Therapists claimed that for those who stay in therapy it works. But the number of those who stay in therapy is very small and there is no systematic evidence for this. As far as the process of the therapy is concerned overall there were no major differences, at least in theory. The overall style of therapy is directive rather than collaborative in Pakistan. Therapy involves a lot of suggestions, advice and support. This could be due to the culturally ingrained value of seeing a person in authority as the source of advice, support and enlightenment. This is in line with Launganis' [13] suggestion that social hierarchies influence the process of the therapy, as well as Iwamasas' [10] observation that "Asian patients like a directive style of therapy". However we have to keep in mind that collaboration does not necessarily mean following strictly a western model of equality of therapist and clients. Every therapy uses techniques which rely on a teacher student like relationship, like educating the patient and use of Socratic dialogue. Similarly, mindfulness based therapy also employs a teacher-student model of therapy.

Therapists reported that behavioural techniques are used commonly and yield good results. This impression could be either due to termination of therapy before the therapist moves on to more sophisticated techniques, or just because the patient wants to get back to work. Probably patients feel less stigmatized and endorsement by the therapist that they can continue to work might make them feel less disabled. The fact that relaxation techniques are found to be helpful only highlight the predominance of anxiety symptoms among patients. Problem solving and building on coping strategies are other helpful techniques and makes perfect sense since majority of the patients present with social or relationship problems. A vast body of literature from Pakistan indicates that depression and anxiety is associated with social and relationship problems in Pakistan [21,22].

Family is not only a part of the assessment but also a part of the therapy. Involvement of the family brings certain strengths but also difficulties into the therapy. Shah et al [23] reported that women living with husbands' extended families were more depressed and anxious than those living in nuclear families. Living with the extended family not only means sharing resources but also the problems. The family can be helpful in improving compliance and follow up as well as in helping the patient in therapeutic work at home.

All the psychologists agreed that psychological help can be useful for patients with psychiatric problems, but the therapy needs to be modified. Unfortunately we were unable to elicit changes being made. However, this study gives us some ideas regarding the strengths and weaknesses in applying therapy in Pakistan.

### Limitations of study

Our study has a number of limitations. We had approached psychologists working in psychiatry departments from only one big city. We mainly focused on issues we had decided to explore. This might have prevented us from discussion of useful information that was not included in our list of ideas to be explored. However, we were careful in our interviews to not ignore any useful cues as they emerged. In order to increase the reliability of the information we not only contacted the interviewees by phone to clarify any issues which arose during the analyses, but we also sent the transcribed interviews to a number of other psychologists to ascertain their level of agreement. We were also unable to interview psychologists with training in CBT. Therapists were trained in REBT. Although there are similarities between CBT and REBT, there are also differences between the two. We also had a small sample size. We stopped interviewing only when we judged that no new themes were emerging. This is however, only initial work and further in depth inquires need to be made to explore these ideas further.

## Conclusions

This is the first study to explore psychologist's experience of providing cognitive therapy to patients in Pakistan. Four major themes emerged on analyses. These were; hurdles in therapy, therapy related issues, involvement of the family and modification in therapy. Psychologists pointed out that therapy needs to be modified to be applied effectively in Pakistan. We were however, unable to obtain detailed information regarding changes that need to be made for therapy to be effective. Factors related to service structure and delivery, patient's knowledge and beliefs about health and the therapy itself were found to have an effect on therapy. While the study was limited by its size, it has revealed useful information concerning the issues to be considered when making cultural adjustments to therapy in Pakistan. It has also clarified areas for further work, identified methodological issues to be addressed and confirmed that more detailed work is necessary to fully explore the barriers and opportunities for improvements in therapies for clients and therapists in Pakistan.

## Competing interests

The authors declare that they have no competing interests.

## Authors' contributions

FN conducted interviews, transcribed and analyzed them. He also wrote the paper. MA and MG analyzed interviews. MG supervised qualitative studies, their analyses and write up. DK supervised the overall project. All authors have read and approved the final manuscript.
